# Dynamic evolution of clonal epialleles revealed by methclone

**DOI:** 10.1186/s13059-014-0472-5

**Published:** 2014-09-27

**Authors:** Sheng Li, Francine Garrett-Bakelman, Alexander E Perl, Selina M Luger, Chao Zhang, Bik L To, Ian D Lewis, Anna L Brown, Richard J D’Andrea, M Elizabeth Ross, Ross Levine, Martin Carroll, Ari Melnick, Christopher E Mason

**Affiliations:** Department of Physiology and Biophysics, Weill Cornell Medical College, New York, NY USA; The HRH Prince Alwaleed Bin Talal Bin Abdulaziz Alsaud Institute for Computational Biomedicine, Weill Cornell Medical College, New York, NY USA; Department of Hematology and Oncology, Weill Cornell Medical College, New York, NY USA; Division of Hematology and Oncology, University of Pennsylvania, Philadelphia, PA USA; Directorate of Haematology, SA Pathology and Department of Haematology, Royal Adelaide Hospital, Adelaide, South Australia; Directorate of Haematology and Centre for Cancer Biology SA Pathology, The Queen Elizabeth Hospital, Woodville, South Australia; School of Pharmacy and Medical Sciences, University of South Australia, Adelaide, South Australia; Feil Family Brain and Mind Research Institute, Weill Cornell Medical College, New York, NY USA; Memorial Sloan-Kettering Cancer Center, New York, NY USA

## Abstract

We describe methclone, a novel method to identify epigenetic loci that harbor large changes in the clonality of their epialleles (epigenetic alleles). Methclone efficiently analyzes genome-wide DNA methylation sequencing data. We quantify the changes using a composition entropy difference calculation and also introduce a new measure of global clonality shift, loci with epiallele shift per million loci covered, which enables comparisons between different samples to gauge overall epiallelic dynamics. Finally, we demonstrate the utility of methclone in capturing functional epiallele shifts in leukemia patients from diagnosis to relapse. Methclone is open-source and freely available at https://code.google.com/p/methclone.

**Electronic supplementary material:**

The online version of this article (doi:10.1186/s13059-014-0472-5) contains supplementary material, which is available to authorized users.

## Background

While deep genetic profiling has revealed striking details about clonal evolution that can drive chemoresistance in cancer progression [1], increasing evidence has shown that epigenetic genes are consistently mutated in many cancers, including glioblastoma [2] and leukemia [3]. These modified epigenetic genes create a new mechanism whereby tumors can evolve and resist therapy, through changes in the epigenetic states, diversity and clonality. Previously, epigenetic polymorphism or ‘epipolymorphism’ has been studied using bisulfite conversion sequencing data [4], which has shown that epigenetic changes can be pervasive across the genome and provide a metric for the overall epigenetic complexity of a sample.

However, the epigenetic clonality of a sample can also be estimated from genome-wide epigenetic profiling methods, such as enhanced reduced representation bisulfite sequencing (eRRBS) [5] or whole-genome bisulfite sequencing (WGBS) [6]. In eRRBS or WGBS data, each read can serve as a representative sample of the epigenetic diversity from bulk cells, since a single sequence read can cover multiple CpGs and simultaneously profile the potential methylation states (C, ^m^C) for all CpGs in that read. Thus, an *epiallele* is a specific DNA methylation pattern of a genetic locus, wherein all CpGs within a single read are effectively ‘phased’ and can represent the epigenetic haplotype (for example, 4 CpGs in one read creates 2^4^ possible patterns, or 16 epialleles). Using these DNA methylation patterns, clonal epigenetic shifts at a given locus can be found by examining the epialleles that change their frequencies. At the global scale, this has been well described before as the epipolymorphism [4]. However, the clonal dynamics of epialleles between different individuals, or from within the same individual, have not been reported before, nor is there an available method by which to discover the sites and types of altered epigenetic clonality.

**Figure 1:**
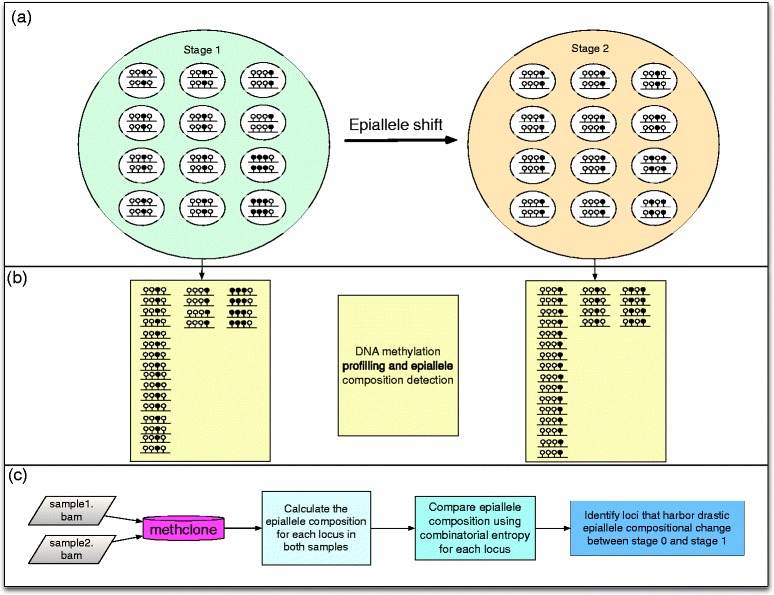
**Epiallele shift detection by methclone. (a)** Schematic plot of epiallele composition of two stages of cells (biggest circles, light green for stage 1 and light brown for stage 2). Each stage has 12 cells with various epiallele compositions. In each cell, there are two lines, represent two set of epiallele at the same locus. Four circles above each line represent four adjacent CpG sites (black: methylated CpG; white: unmethylated CpG). **(b)** Bisulfite conversion sequencing output reads that spanning at least four CpG sites will capture the epiallele composition at each stage. **(c)** methclone workflow. methclone take the bam file from Bismark to calculate the epiallele composition and compare them from different samples. In the application of the combinatorial entropy, methclone determine the loci that harbor significant epiallele compositional change.

To address this challenge, we have developed a novel, open-source algorithm and freely available set of analysis tools collectively called *methclone* [7] that can discover and annotate epigenetic loci (eloci) that have a large compositional change of clonal epialleles between two different stages. Methclone calculates the combinatorial entropy (ΔS) change of epialleles at one locus and outputs the loci with a ranked list of epiallele changes defined by the entropy change (Figure 1), from no change (0) to maximum difference in entropy (-144). These ranked epialleles can be easily integrated with other published tools for DNA methylation alignment, QC, and annotation such as methylKit [8] and eDMR [9]. Using methclone, we found thousands of loci across the genome harbor significant (∆S < -70) changes in their epialleles, and we found that these occur in genes critical for cell regulation and cancer development, including SOX2, SOX9, ERBB2, and BMP1. Moreover, we show that our metric of epiallele shifts per million loci (EPM) is a normalized measure of a sample’s global epiallele clonality that can allow a comparison between different samples and reveal samples with dramatic changes in a sample’s overall epigenetic landscape. Taken together, these methods create a novel, rapid means by which to detect, trace, and prioritize genomic areas with shifts in their cells’ epigenetic states and can be used to define epiallelic clonality, tumor evolution, and epigenome dynamics.

## Results and discussion

### Detection of significant epiallele shift between different stages of tumor

**Figure 2:**
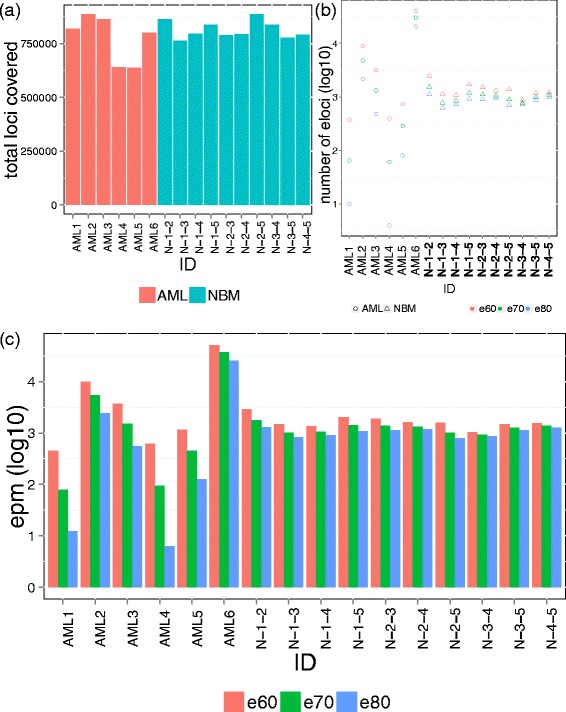
**The total number of loci and eloci. (a)** The total number of loci composed by four adjacent CpG sites was plotted for each patient. **(b)** The number of eloci (log 10 transformed) for each patient (pair-wise comparison between diagnosis and relapse) or between normal bone marrow samples using three different combinatorial entropy difference cutoffs (e80: -80, e70: -70, e60: -60). **(c)** The value EPM (log 10 transformed) for each patient (pair-wise comparison between diagnosis and relapse) or between normal bone marrow samples using three different combinatorial entropy difference cutoffs (e80: -80, e70: -70, e60: -60).

One large challenge in understanding cancer is the cellular basis of relapse, wherein patients treated at diagnosis with chemotherapy often relapse with a more aggressive disease within a few months or years. When the disease returns, the cells may or may not carry the same genetic or epigenetic background, compared to the status at diagnosis [5]. To understand this problem at the level of the epialleles, we obtained samples from six patients with acute myeloid leukemia (AML), who were first diagnosed as primary AML and reached first complete remission before presenting with relapsed AML (Additional file 1: Table S1). Enhanced reduced representation bisulfite conversion sequencing (eRRBS) was performed to obtain the DNA methylation status in the CpG-enriched regions across the genome, using standard protocols [5]. We first calculated the methylation pattern from the same location defined by four adjacent CpGs covered by the same read, with at least 60 reads covered for each patient’s bisulfite conversion sequencing data. On average, we observed 773,350 loci covered by both stages of samples for each patient (S.D. = 108,514, Figure 2a).

**Figure 3:**
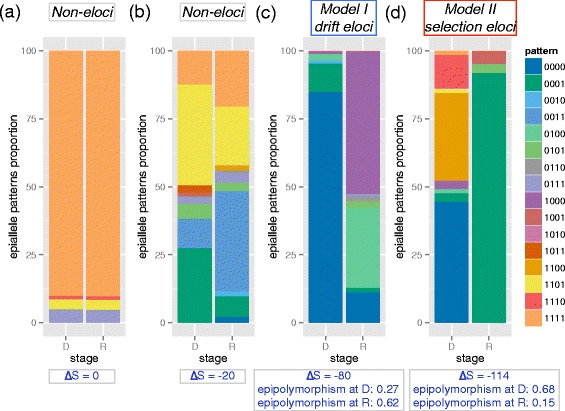
**Loci with and without significant epiallele shift.** Epiallele pattern proportion change on non-eloci (**a**, Δ$\mathrm{S}$ = 0, chr1: 234367244-234367265, SLC35F3 exon), non-eloci (**b**, Δ$\mathrm{S}$ = -20, chr1: 103319658-103319682, downstream of COL11A1 gene), drift eloci (**c**, model II, Δ$\mathrm{S}$ = -80, chr16: 330195-330217, epipolymorphism at diagnosis: 0.27, at relapse: 0.62, increased by 0. 35, ARHGDIG promoter) and selection eloci (**d**, model I, Δ$\mathrm{S}$ = -114 , epipolymorphism at diagnosis:0.68, at relapse: 0.15, decreased by 0. 53, chr10: 109674359- 109674402, SORCS1 distal upstream). Model I is defined as eloci with increased epigenetic tumor heterogeneity measured by epipolymorphism. Model II is defined as eloci with decreased epigenetic tumor heterogeneity measured by epipolymorphism.

Next, we compared the epiallele composition between diagnosis (D) and relapse (R) stages for six AML patients (Additional file 1: Table S1). The epiallele composition and percentages were calculated for each covered locus, comparing the D vs. R status within each patient separately (Figure 3). All patients’ cells were processed at the same facility with the same purification methods (Ficoll gradient separation of mononuclear cells followed by lymphocyte depletion using CD3 and CD19 Miltenyi bead negative selection). Using the combinatorial entropy (as defined above), we examined the epiallele composition at each locus. The combinatorial entropy ranges from 0 to -144. The lower the combinatorial entropy, the larger the difference in epiallele composition between stages.

Methclone can also give a quantification of the changing eloci, estimate their mode, and help visualize them. This can be important for eloci with a large clonal change, such as those between diagnosis and relapsed stages. We used the 16 colors to define the 16 patterns of epialleles (Figure 3) at four loci, where ‘0’ stands for an un-methylated CpG and ‘1’ stands for a methylated CpG. Figure 3a shows a location with the fully methylated epiallele as the major pattern at both stages, with less than 10% of reads supporting the other three patterns (combinatorial entropy = 0). The second locus has some heterogeneous epialleles at each stage, but generally maintained the epiallele spectrum at relapse stage, with combinatorial entropy = -20 (Figure 3b). For these samples, we found an average of 99.2% (S.D. = 0.10%) of their epialleles are sustained after treatment, defined as having combinatorial entropy from 0 to -20 (Figure 3a and b).

However, for other loci in more dynamic patients (for example, AML2 or AML6), the epiallele composition showed a much larger change (combinatorial entropies are -80 and -114, respectively). For example, the third locus in ARHGDIG promoter at the diagnosis stage was mainly (85%) composed of epialleles ‘0000’ and 11% of ‘0001’, but changed to 53% of ‘1000’ at relapse stage, and other patterns including ‘0100’ (29%) and ‘0000’ (11%) (Figure 3c) also showed large shifts. Since the global epigenetic heterogeneity as measured by epipolymorphism increased from the diagnostic stage (0.27) to the relapse stage (0.62), we propose this epiallele shift is represented by a drift model (Figure 3c).

Further, in the fourth locus in SORCS1 at the diagnosis stage (Figure 3d), the major epialleles are ‘0000’ (45%), ‘1100’ (32%). At relapse, the major epialleles changed dramatically to ‘0001’ (3% at diagnosis to 92% at relapse), which represents a large epialleie shift at ∆S = -114. Also, the epipolymorphism at diagnosis is 0.68, but decreased at relapse stage to 0.15. Given this large epiallele shift, concomitant with decreased epigenetic heterogeneity at relapse, and we define this type of epiallele shift as the putative selection model (II). As noted here, the differences in the epipolymorphism at loci can increase or decrease, yet mask the change in level of clonality. However, the ∆S can provide this metric on a linear scale, and it also contextualizes the different directions of altered epipolymoprhism (increase and decrease) into two proposed models (drift vs. selection).

### Tumor cells undergo genome-wide, significant epiallele compositional change after treatment

We then quantified the number of loci that undergo significant epiallele shift (eloci) by using three entropy cutoffs (e: -80, -70, and -60). The total number of loci covered by sequencing is similar among samples (Figure 2a). The number of eloci decreases with more stringent cutoffs (Figure 2b); we observed that the epiallele eloci were widely varied for different patients at the same cutoff. Specifically, patient AML6 had the highest number of eloci (n = 40,361 at e60), and this was true regardless of the entropy cutoff (Figure 2b). Also, one patient (AML3) showed a moderate number of eloci, with 3,163 at e60. But, the lowest amount of eloci were observed in AML1, AML4, and AML5, with on average 498 eloci at e60. We then normalized the number of eloci from each patient relative to the total number of covered CpGs, to ensure it is not affected by the total number of covered loci from the ERRBS data; this creates an estimate of the eloci per million CpGs covered (EPM, see methods). The number of eloci, though, was not significantly correlated with the total number of loci, or sequencing library depth (Additional file 1: Figure S1). After normalization of the total number of covered loci by sequencing, AML3 still showed the highest EPM among all five patients, indicating that the degree of epiallelic change is indeed highest in this patient (Figure 2c).

To ensure measurement accuracy, we performed an additional library preparation, and sequencing of one of our AML samples (AML6). We found that the replicate’s DNA methylation levels for all CpGs showed a high R^2^ (0.96, Additional file 1: Figure S1a), confirming previous reports showing that variation of the DNA methylation is mostly not due to the variability in the ERRBS process itself [5]. We then applied methclone to both technical replicates, in order to examine the technical noise behind our epigenetic measures. We found that the largest delta combinatorial entropy (ΔS) from technical replicates is -32, and the mean is -2, which led to no significant epiallele shift being detected between these technique replicates (Additional file 1: Figure S1b). This also gives an upper bound of technical variation for our epiallele change metrics, which is well below thresholds used by default (-60), and shows evidence of very low technical variation.

To further contextualize the variance of epialleles and EPM shifts in these leukemia patients relative to controls, we calculated the EPM for five normal bone marrow samples (Figure 2). We show that methclone can also capture inter-individual epiallele differences. We did pairwise comparison between five NBM samples, in total of 10 comparisons. We observed a more stable number of eloci captured between NBM samples, likely representing the average inter-person epigenetic individuality at the same loci from the same tissue. On the other hand, the number of eloci between the same individual at different stages of AML actaully showed more diverse values of EPM (sd > 10,200 for AML, sd < 498 for NBM, for all three cutoffs). Interestingly, this indicates that the number of different epialleles (eloci) between unrelated individuals are rather stable, whereas the number of eloci are much more dynamic within the same person during a tumor’s treatment and progression.

### Genome annotation and distribution of eloci

**Figure 4:**
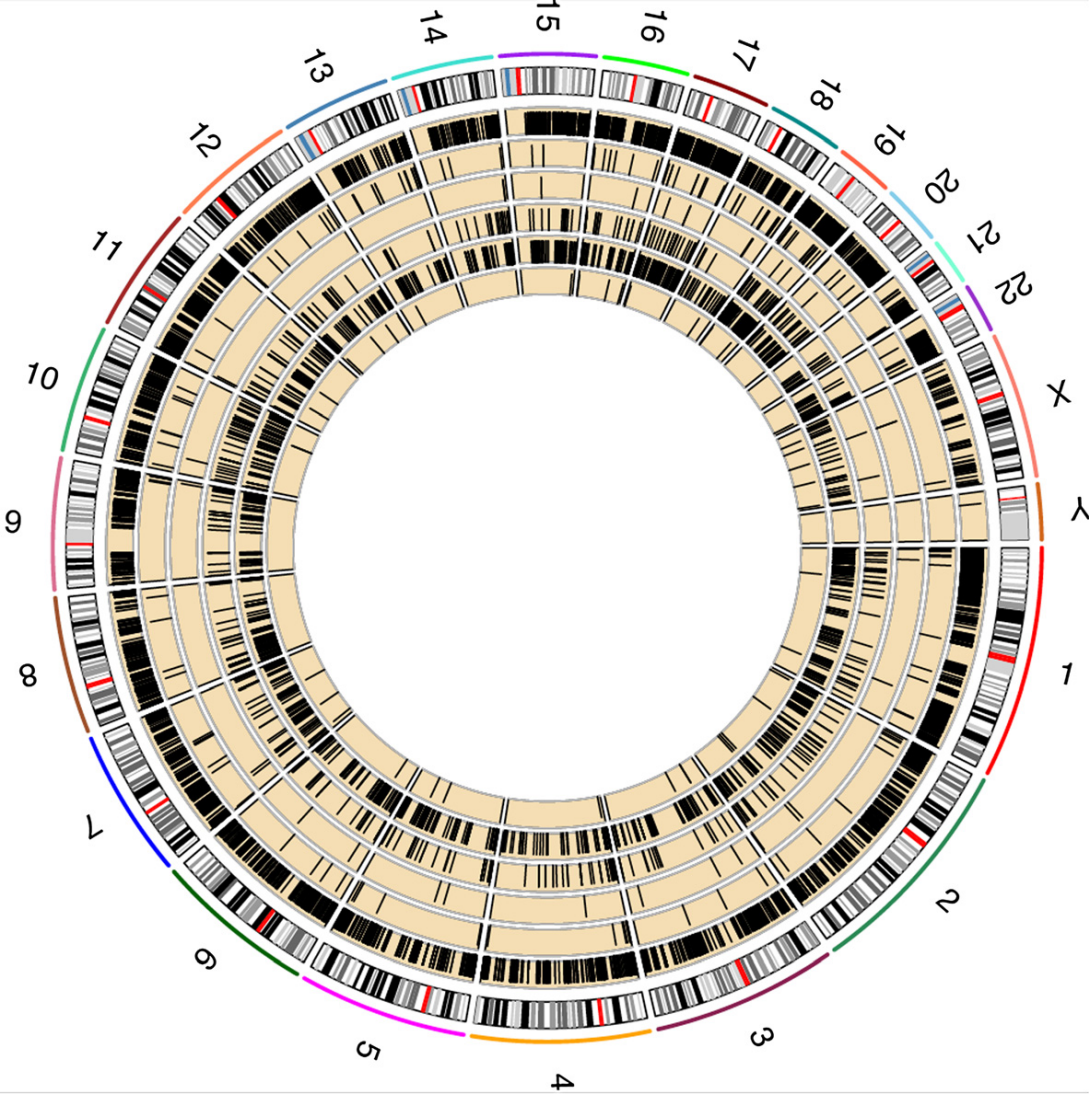
**Global epiallele pattern reconstruction between diagnosis and relapse stages.** The number at the outer track is the chromosome number. The next track is ideogram of hg19. The five yellow tracks are AML1-6 from inwardly. Black bar in each yellow track stands for eloci determined using -70 as combinatorial entropy cutoff.

**Figure 5:**
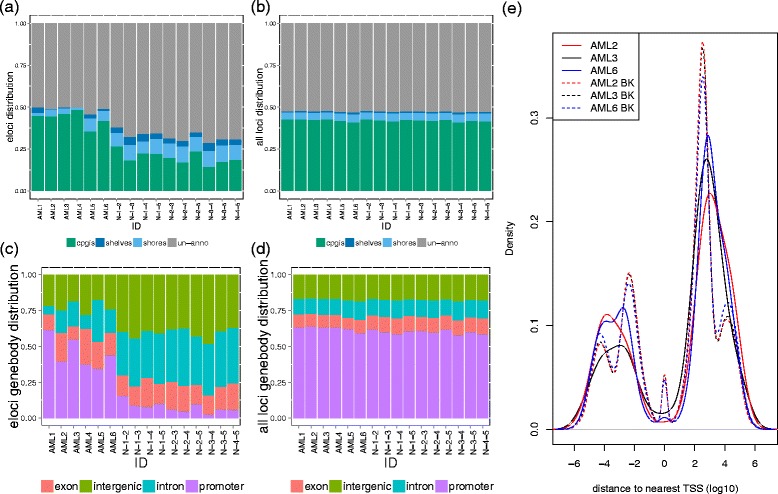
**Genome distribution of the eloci during leukemia progression. (a, b)** The distribution of eloci **(a)** and background loci **(b)** in CpG islands, shores, shelves, and the rest of the genome. **(c,** **d)** The distribution of eloci **(c)** and background loci **(d)** in promoter, exon, intron of RefSeq gene model and intergenic regions. **(a-** **d)** eloci from pairwise comparison of five NBM were also plotted to compare and contrast with the eloci during leukemia progression. **(e)** The density plot of the distances of the eloci (solid lines) and background loci (BK: dashed lines) to the nearest transcription starting sites (TSS) in log10 scale for AML2 (red), AML3 (black), and AML6 (blue).

The epiallele patterns between stages were observed as global and widespread events (Figure 4). To demonstrate this, we created a circos plot of the patterning for the epiallele dynamics of all six AML patients in five tracks inside the ideogram of hg19. Eloci from AML1 to AML6 were plotted outwardly. To determine region-specific enrichment of eloci relative to CpG density, we mapped all eloci to annotated CpG islands. We showed that the eloci distribution was dramatically different for each patient across the CpG islands (cpgis), shores (2 kb regions at each side of CpG islands), shelves (2 kb regions at left side of left shore and right side of right shores), or un-annotated areas (location outside of all above regions) of the genome (Figure 5). On average, 43% of the eloci were located in the CpG islands, with a smaller portion of them distributed in the shores (4%) and shelves (1%). Thus, 51% of eloci were located outside of these CpG-annotated regions, which indicates that a large fraction of non-promoter, dynamic epigenetic changes are occurring in these samples, matching results previously observed in leukemia [5]. The eloci from NBM samples showed lower enrichment of CpG islands (20%), but higher enrichment in shores (9%) and shelves (4%), which indicate a different pattern than eloci from AML samples. As a control, we confirmed that the background distributions of covered loci from AML and NBM samples over genes all have similar distribution (Figure 5b and d).

We then further examined the distribution of eloci as a function of their proximity to genes (Figure 5) using all current RefSeq gene models (v66). We observed that the majority of the eloci were distributed in the genic regions (promoter, intron, exon, Figure 5c), with an average of 45% of eloci located in the promoter regions (background: 63%), 16% located in exons (background: 8%) and 15% in introns (background: 12%). Interestingly, the sites of dynamic changes are enriched for intronic areas (up to 29%) in AML5, but other samples can have enrichments for other genomic areas such as exons (for example, AML2,4-6). This indicates that *both* the specific sites of eloci and their global enrichment can change within a patient over time. While those loci from pairwise comparisons of NBM samples showed depletion from promoter regions (8%) and enrichment in intron (36%) and intergenic (41%) regions, the background distribution of covered sites is similar between AML and NBM samples. We further examined the eloci of NBMs for enrichment of enhancers (Broad institute chromHMM), and found no enrichment for strong or weak enhancers (Additional file 1: Figure S3). Rather, we observed that two tumor samples’ eloci were completely depleted of enhancer (or strong enhancer) marks (AML3,4), again indicating a larger degree of re-distribution of dynamic epialles in the tumor samples compared to the NBMs.

We next examined the distance of eloci to the nearest transcriptional start site (TSS) from RefSeq gene models, plotting the location upstream of TSS (negative value) to downstream of TSS (positive value) against the density of eloci (Figure 5e). We observed lower density of eloci around the TSS for all samples, which indicates that the DNA methylation levels at the TSS sites are less dynamic than other regions upstream and downstream. A higher density of eloci was observed downstream of the TSS, which matches the proportion of eloci that are located in the genebody (exon and introns), and notably, these were significantly different between different tumor samples (AML3 vs. AML6, *P* value = 0.00278, Wilcoxon rank sum test).

**Figure 6:**
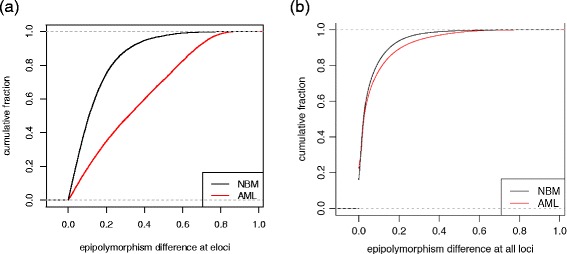
**Higher epigenetic heterogeneity changes observed in loci with significant epiallele shift in AML, compared to those from NBM. (a)** Significantly higher absolute epipolymorphism difference at eloci between AML pairs than eloci from pairwise comparison of five NBM samples (*P* value <10-16, Wilcoxon rank sum test with continuity correction). **(b)** Cumulative fraction of absolute epipolymorphism difference for all loci covered in AML samples and NBM samples.

Finally, we compared the epigenetic heterogeneity changes in eloci from AML or NBM samples (Figure 6). Since there is no specific direction for comparison between NBM samples (similar to D vs. R in AML), we measured the absolute difference in epipolymorphism at the dynamic epiallelic sites found by methclone. As expected, we found higher epigenetic heterogeneity in loci with significant epiallele shifts in AML, compared to those from NBM (Figure 6). This indicates that the eloci selected between AML patients at different diagnosis and relapse stages have undergone a larger extent of selection, drift, or depletion of their epigenetic states. We then further functionally annotated the two sets of AML and NBM eloci with GREAT [10] functional gene annotations. We found that NBM eloci do not enrich in any pathways or gene ontology terms, while AML eloci were enriched in: transcription regulatory region DNA binding, transcription factor binding, regulatory region DNA binding, chromatin binding (GO molecular function); cell-cell signaling, cell development, cell fate commitment (GO biological process); cancer (Disease ontology); Wnt signaling pathway, cadherin signaling pathway, focal adhesion (PANTHER pathway), and MAPK signaling pathway (MSigDB pathway). These enrichments indicate that the clonal changes in epigenetic states contribute to disrupted pathways in leukemia and contribute to the relapsed state.

## Conclusion

Dysregulation of DNA methylation or their controlling genes has been established as a hallmark of certain cancers [5,11]. These epigenetic changes impact the biological activity of cells through their modification of transcriptional states and regulatory machinery, and the proportion of cells carrying these mutations is known to vary at the genetic level [12,13]. But, a method for determining the proportion of cells that carry these changes has never been demonstrated at the epigenetic level, even though it has been reported that a subset of the CpG sites vary their methylation level between 20% and 80% [8]. Indeed, the epipolymorphism measure was the first specification that could demonstrate and quantify intra-sample heterogeneity [4]. However, the change in epipolymorphism of a sample or locus does not reveal the underlying clonality. For example, given a locus with four adjacent CpG sites with similar average methylation levels, the epipolymorphism may change only slightly (0.05), whereas the composition of the epiallele pattern could significantly shift for one or more epialleles (ΔS -80). Given two samples with comparable epipolymorphism, the epiallele composition at specific sites could be completely different, and only by tracking the epialleles can one discern the clonal shifts.

Using methclone, we were able to measure widespread intra-patient epiallele dynamics in AML patients between diagnosis and relapse. Our approach locates and quantifies the degree of epiallele compositional changes that would not captured by global epipolymorpism, as global heterogeneity can stay the same even when specific epialleles’ compositions are completely changed. For example, the ΔS gives a wider dynamic range than epipolymophism (0 to -144 vs. 0 to 1) and also reveals sub-clones that are not apparent by a general epipolymoprhism measure (Figure 3). Also, our combinatorial entropy metric (ΔS) can reveal and quantify changes that would be missed by traditional entropy measures such as Hamming distance [14] (Additional file 1: Figure S4), indicating a more comprehensive measure of epigenetic change. Therefore, the epiallele dynamics measured with Methclone creates a novel means by which the clonal selection and shift of epialleles can be discovered, quantified, and prioritized.

In this novel application of the combinatorial entropy calculation [15,16], we were able to measure the extent of epiallele compositional change between two stages. Using leukemia samples at diagnosis and relapse stages as a model, we have determined that methclone can discern the dynamic loci (eloci) that harbor significant epiallelic shifts, and these eloci are widespread across the genome. The connection (if any) between the chemotherapy received, the status of the patient’s intrinsic folate metabolism and the epiallelic shift observed upon relapse remains to be investigated, but is now approachable. Indeed, *methclone* revealed that tumors can show a unique profile of both the sites of epiallelic shift as well as their distribution across the genome. We demonstrated the epiallele shift detected between different stages of AML is fundamentally different from that between randomly selected normal bone marrow. NBM samples are depleted from gene promoter and CpG islands compared to those from AML samples (Figure 5), and the eloci from AML samples significantly enriched for Wnt pathways, MAPK signaling pathways, ontology in cancer, and transcription regulatory regions sequence specific DNA binding. These sites and global measures can be quantified as eloci per million loci covered (EPM), and this measure, along with epipolymorphism, could potentially be used as a new measure of the overall degree of epigenetic dysregulation in a sample.

Since there are at least two putative means (Figure 3c and d) by which epialleles can shift between two samples or time points, we estimate both types with our algorithm. The first model is that drift DNA methylation changes led to increase of epipolymorphism, caused by a stochastic or directed DNA methylation process, such as those mediated by DNA methyltransferases (DNMTs), proceeding in a step-wise fashion. The second model is that an enrichment of an epiallele emerges as bulk cells undergoing selection during chemotherapy or treatment, leading to a decrease in epigenetic heterogeneity.

However, the epiallele dynamics at two time points/stages may indicate important regions involved in these biological processes, and our algorithm attempts to discern the two models by flagging those epialleles that are drift versus selection and those which do not change in epigenetic heterogeneity.

Although this work is focused on DNA methylation, these methods would be germane to any base-modification of DNA (epigenome) or RNA (epitranscritpome) with single-base resolution data and multiple sites assayed within the same sequencing read [17,18]. Notably, our utilization of combinatorial entropy (ΔS) as a measure of epiallelic shift allows us to gauge the difference of the epiallele composition from the same set of epialleles, as well as the epiallele changes occurring from an emergence of new epiallele. Future work could create a more deliberate approaches to measure greater inter-sample epiallele shift (more epialleles), where longer reads are used, or samples could also undergo an enrichment (capture) of particular epialleles to get improved assessment of clonality. Also, the general differences between these epialleles in different tissues from the same individual have not yet been established, and data to understand the baseline epigenetic shifts in normal individuals have only recently begun to be gathered [19]. Nonetheless, the success of measuring the epiallelic shift in leukemia has already shown promise in serving as an estimate of the evolutionary and clonal distance between stages of diseases or development, thus expanding our knowledge of epigenetic heterogeneity and how epialleles can change within a patient, over time, across the genome.

## Materials

### Data source and preprocessing

Six acute myeloid leukemia (AML) de-identified patient samples at two time points (diagnosis and relapse stages), enriched for myeloblast cells, were used in the experiments, and these samples were also compared to five CD34+ normal bone marrow samples (AllCells, Inc.). Institutional review board approval was obtained at Weill Cornell Medical College (IRB # 0805009783) and at the Royal Adelaide Hospital, and this study was performed in accordance with the Helsinki protocols. DNA was extracted using standard techniques and ERRBS library preparations were performed as previously described [5]. Libraries were sequenced on a HiSeq2500 Illumina machine using 75 bp single-end reads to an average depth of 74× per covered CpG. We performed bisulfite treated read alignment to hg19 genome and methylation calls as previously described [5]. Briefly, the adaptor sequences in the raw Illumina reads were removed using FAR software. Then Bismark aligner was applied for mapping of the preprocessed reads to human genome, with only uniquely mapped reads kept for DNA methylation calling. Because CHH and CHG sites should be all unmethylated, we then calculate the C- > T conversion rate by calculating the average percentage of reads support T among all the reads support T and C in CHH and CHG sites. We confirmed that the conversion rate for all samples was at least 99.8%, as previously described [9].

### Data deposition statement

All data have been deposited for public access in the dbGap database. The accession number is phs000793.v1.p1 [20].

### Definition of the algorithm of methclone

The methclone algorithm tests the hypothesis of significant epiallelic shift. In order to find the optimized number of CpG sites to define the epiallele, we calculated the number CpG sites covered by each read, up to 10 CpGs (Additional file 1: Figure S5a). We then plotted the number of patterns of epiallele *n* against the number of CpG sites to define epiallele ‘*x’* (Additional file 1: Figure S5b), where *n* = 2^*x*^. The read count per epiallele were then plotted against CpG number to define epiallele (4 - 10), which shows a large drop in the read count per epiallele when changing from four CpGs to five CpGs (Additional file 1: Figure S5c). The higher the number of CpGs that is used to define one locus, the higher the read number needed to measure the full spectrum of epiallele patterns. Therefore, if one loci defined by *x* adjacent CpG sites use *m* covered reads to calculate 2^*x*^ epiallele patterns composition, then loci defined by *x* + 1 adjacent CpG sites will use 2 × *m* covered reads to cover 2^*x* + 1^ epiallele patterns composition. This further increase the large drop in the capacity of measurement between epialleles defined by four CpGs or five CpGs. In order to have a good coverage for each locus and obtain a full spectrum of epiallele patterns and wider extent of genomic loci, we choose four CpG sites to define epialleles in these samples. However, this is a modular option for the algorithm, which can be increased to find more epialleles given a longer read length.

All 16 possible patterns for epiallele of four adjacent CpG sites are shown in Additional file 1: Figure S6. If one epiallele has been enriched from one stage to another, then the region harbors an epiallele shift that may lead to a drift model or selection model (Figure 3c and d). Before these two models can be distinguished, a means to find those sites with the largest change must first be established. To do this, *methclone* compares the prevalence of the epiallele patterns at one group to another the full set of all covered loci. The bigger the difference in the epiallele patterns composition, the more likely it is a hotspot that harbors epiallele shift.

### Foreground combinatorial entropy

In order to quantify the degree of epiallelic shift, we applied combinatorial entropy [15,16,21], a measure to compare the difference distributions of the epialleles between different stages. The estimated change between stage *k* = 1 and stage *k* = 2 at locus *j* were defined by *S*_*j*_, which is the sum of the nature logarithm transformation of the total number of permutations *Z*_*k*,*j*_:

1 $$ {S}_j={\displaystyle \sum_{k=1,2}}{S}_{k,j}={\displaystyle \sum_{k=1,2}} \ln {Z}_{k,j}={\displaystyle \sum_{k=1,2}} \ln \frac{N_{k,j}!}{{\displaystyle {\prod}_{i=1,\dots, 16}}{N}_{i,k,j}!} $$

where *N*_*k,j*_ is the total number of epialleles at stage *k* at locus *j*. The read count of epialleles was normalized by the sequencing library size. We then normalized the total number of reads covering each locus to be constant (200) to control the entropy dynamic range. *N*_*i,k,j*_ is the total number of epiallele for pattern *i* in stage *k* at locus *j*. This is calculated using *N*_*k,j*_ times the percentage of reads support pattern *i* in satge *k* at locus *j*. When four adjacent CpGs define locus *j*, the number of patterns in total is 16. In the combinatorial formula, the total number of permutations of *N*_*k,j*_ epialleles is divided by the product of the number of indistinguishable permutations for each epiallele pattern *i* (where there are *N*_1,*k*,*j*_ indistinguishable read support epiallele *1*, *N*_2,*k*,*j*_ indistinguishable read support epiallele *2*, …, and *N*_16,*k*,*j*_ indistinguishable read support epiallele *16*). When each stage only has one pattern, the entropy *S*_*j*_ is equal to zero (for example, all four adjacent CpGs unmethylated or all methylated). When all 16 epiallele patterns exist with a uniform distribution at each stage, the entropy *S*_*j*_ is maximal.

### Background combinatorial entropy

The background epialleles distribution is defined when all patterns of epialleles are uniformly mixed between the two stages. For locus *j*, one can compute the combinatorial entropy *S*_*j*_, as defined by Equation 1 (above). *S*_*j*_ with fixed epialleles across stages has a maximal value given by the background entropy ${\overset{\sim }{S}}_i$ for uniformly distributed epialleles,

2 $$ {\tilde{S}}_j={\displaystyle \sum_{k=1,2}}{\tilde{S}}_{k,j}={\displaystyle \sum_{k=1,2}} \ln \operatorname{}{\tilde{Z}}_{k,j}={\displaystyle \sum_{k=1,2}} \ln \frac{N_{k,j}!}{{\displaystyle {\prod}_{i=1,\dots, 16}}{\tilde{N}}_{i,k,j}!} $$

where ${\overset{\sim }{N}}_{i,k,j}$ is the expected epialelles for pattern *i* in the locus *j* of the stage *k*, provided that all the epiallele patterns are uniformly distributed across stages:

3 $$ {\tilde{N}}_{i,k,j}=\frac{N_{k,j}{N}_{i,j}}{N_j} $$

Here *N*_*i*,*j*_ is the total number of epiallelwith pattern *i* in locus *j* across two stages. *N*_*j*_ is the total number of epialles in locus *j*.

### Eloci definition and epiallele patterns reconstruction measurement

We define eloci as those that have different distributions of epialleles between two stages. Using the entropy difference *ΔS*_*j*_ between foreground and background combinatorial entropy, one can quantify the degree to which the composition of epialleles at a given loci *j* are distinctly different between two stages. The lower the value of *ΔS*_*j*_, the mo different between stages. The locus *j* is defined as elocus if:

4 $$ \Delta {S}_j={S}_j-{\tilde{S}}_j < \alpha $$

Where *α* is the cutoff to determine if the entropy difference is large enough to discern whether the epiallele patterns’ distribution has a significant change between two methylomes. *ΔS*_*j*_ ranges from 0 to -144. The distribution of *∆S* over the average read coverage (Additional file 1: Figure S7) and average DNA methylation (Additional file 1: Figure S8) shows that there is no strong correlation. We performed the analysis using a range of values *α* from -60 to -80 with a decrement of 10. The result reported by methclone is a list of eloci and the corresponding epiallele patterns distribution.

To compare the epiallelic loci (eloci) between samples with various coverage, we also defined a variable *EPM* (the number of eloci changed per million loci covered by sequencing data) to measure the overall epiallele pattern reconstruction between two stages as

5 $$ EPM=\frac{10^6}{C}\times E $$

Where *E* is the total number of eloci detected between different stages, *C* is the total number loci covered by both samples.

### Epipolymorphism calculation

Epipolymorphism was calculated as previously described by Landan *et al.* [4]. Epipolymorphism has been used to measure epigenetic heterogeneity [22], but it does not represent the epiallelic shift between samples. For each loci, the epipolymorphism $e=1-{\displaystyle \sum_{i=1}^{16}}{p}_i^2$, where *p*_*i*_ is the percentage of *i*th epiallele.

### Hamming distance for epiallele shift detection

Hamming distance can measure the diversity between two strings with fixed length by calculating the number of site at which the corresponding symbols differ. We implemented hamming distance here for each locus of four adjacent CpGs, where the symbols are 16 different epiallele patterns. The cutoff for the existence of the pattern is 5% at each stage. Although the correlation between the hamming distance and the entropy are very high (Pearson correlation: 0.84, Additional file 1: Figure S4), there are cases where the hamming distance is high, for example, when the hamming distance is five, there are more than five epiallele patterns, and the entropy range is from -20 to -110. So, these data show how the combinatorial entropy difference can affect differences that are masked by the Hamming quotient, through leveraging the information of the epiallele proportion.

However, to make Methclone more comprehensive, we also supplied a script to calculate the hamming distances between the major epiallele patterns of two samples/stages, as well as using the existence of epialelle patterns that have more than 5% as the symbols in the hamming distance. This can be found here: [23], as well as other useful scripts that interface with the code. Interestingly, we see that there is an overall similar trend of increasing Hamming distance revealing greater epiallele shift (Additional file 1: Figure S4), but most importantly, there are clear cases where the hamming distance is the same, yet the clonal architecture is dramatically different. We believe these additional data point to the limited dynamic range of a Hamming discrete count, but if other algorithms become available that use Hamming distances in other ways, we can easily modify our scripts to accommodate these other methods (as here).

### Single nucleotide polymorphism calculation

We applied Bis-SNP [24] to detect single nucleotide polymorphisms in the ERRBS data and removed any loci that overlap with C > T or G > A SNPs. We have provided and example on our website of how to remove any sites that show genetic variation during an analysis, should a user wish to ensure common variants are not impacting their measurement of epialleles or to partition their analysis. See [25].

### Genomic locations annotation

RefSeq gene model and CpG islands track were download from UCSC genome browser website and were used for loci and eloci annotation with GenomicRanges [26]. Circos plot were plotted using RCircos [27].

### Pathway enrichment analysis

Pathway enrichment analysis was performed using the GREAT [10] Great associates genomic regions with nearby genes’ regulatory domains. Specifically, we used the following parameters for the definition of regulatory domain: 5,000 bp upstream, 1,000 bp downstream of TSS as basal regulatory domain and this is extended up to 50 kb maximum. These genes then were included to calculate enrichment statistics using the binomial test and the hypergeometric test. Only the gene ontology terms and pathways significant by both tests are included (FDR q-value <0.01).

## Additional file

Additional file 1: Figure S1. The number of eloci against the number of loci covered (a) and sequencing library size (b). **Figure S2.** Technical reproducibility of RRBS and ∆S. **Figure S3.** No enrichment for enhancer for eloci from NBM samples. **Figure S4.** Hamming distance vs. delta combinatorial entropy (ΔS). **Figure S5.** Coverage of CpGs by sequencing data. **Figure S6.** Sixteen patterns of epialleles defined by four CpGs within one read. **Figure S7.** Average read coverage against delta combinatorial entropy. **Figure S8.** Average DNA methylation against delta combinatorial entropy. **Table S1.** Clinical information for AML patients.
