# Supplementary material for: Dynamic evolution of clonal epialleles revealed by methclone
Source: Genome Biol. 2014 Sep 27;15(9):472. doi: 10.1186/s13059-014-0472-5 (PMC4242486; doi:10.1186/s13059-014-0472-5)
Supplement: Additional file 1: Figure S1. — The number of eloci against the number of loci covered (a) and sequencing library size (b). Figure S2. Technical reproducibility of RRBS and ∆S. Figure S3. No enrichment for enhancer for eloci from NBM samples. Figure S4. Hamming distance vs. delta combinatorial entropy (ΔS). Figure S5. Coverage of CpGs by sequencing data. Figure S6. Sixteen patterns of epialleles defined by four CpGs within one read. Figure S7. Average read coverage against delta combinatorial entropy. Figure S8. Average DNA methylation against delta combinatorial entropy. Table S1. Clinical information for AML patients. [file 13059_2014_472_MOESM1_ESM.pdf]

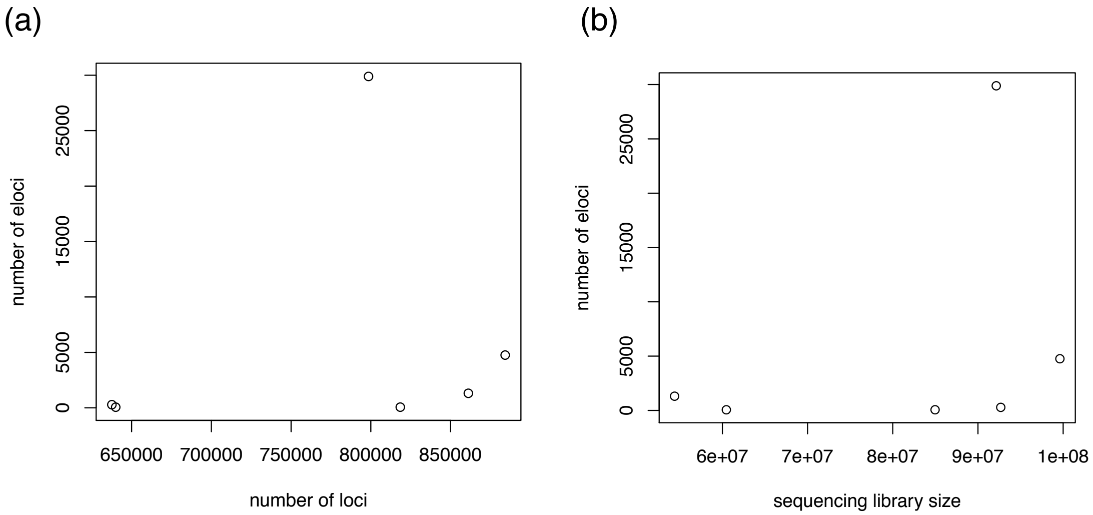

Figure S1. The number of eloci against the number of loci covered (a) and sequencing library size (b). The correlation is 0.21 (a) and 0.36 (b). Both p-values for the test of Pearson correlation is greater than 0.1. ΔS cutoff is -70.

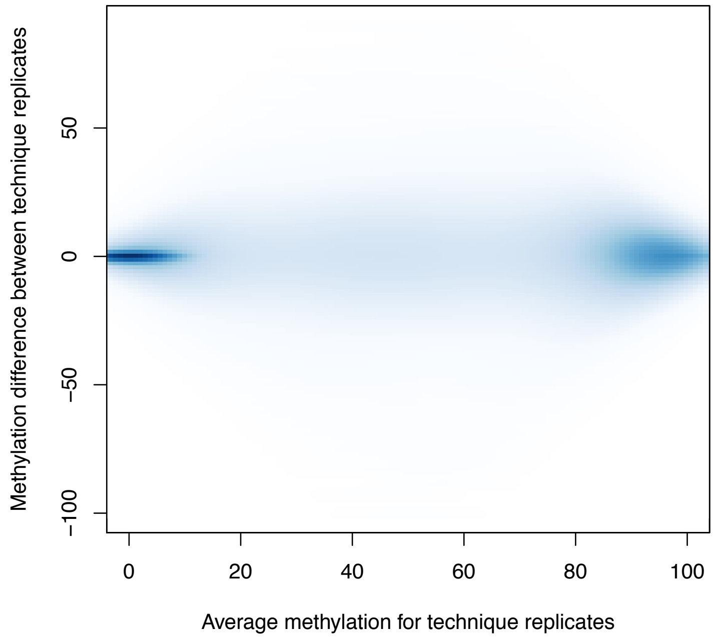

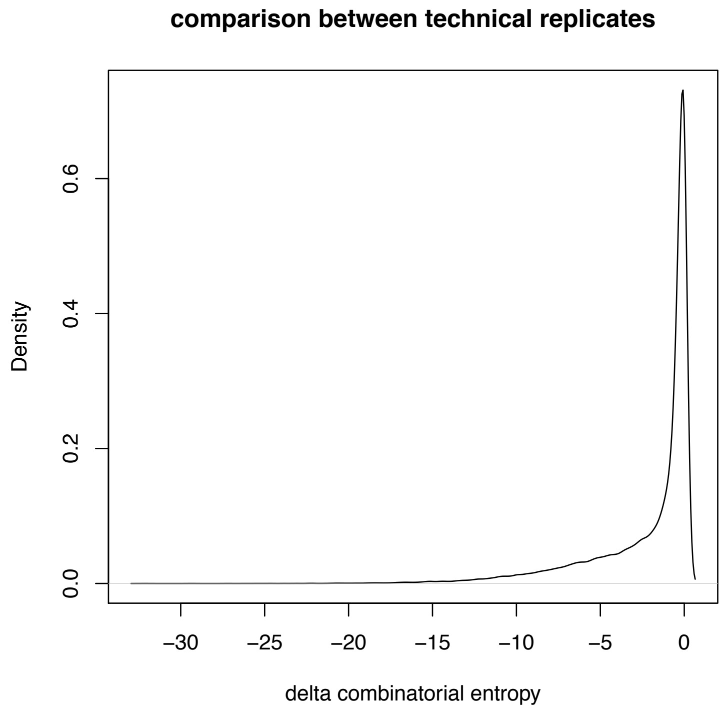

Figure S2. Technical reproducibility of RRBS and Δ S. (top) Methylation difference against average methylation between technique replicates for AML6, where we observed very high concordance of methylation measures (R^2=0.98). (bottom) The density plot of the delta combinatorial entropy between two technical replicates of sample AML6.

(a) (b)

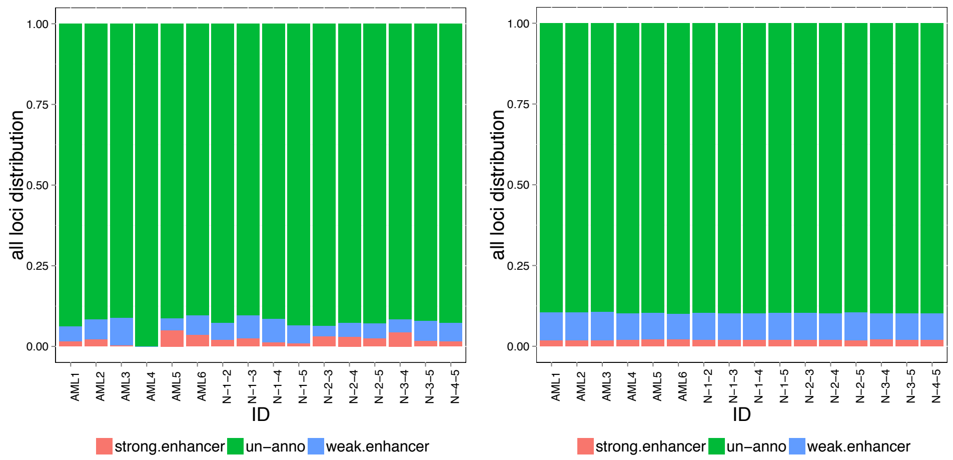

Figure S3. No enrichment for Enhancer for eloci from NBM samples. We plotted the proportion of eloci that show enrichment for strong enhancers (red), weak enhancers (blue), or un-annotated areas of the intergenic sites (green). (a) For the AML and NBM samples, we observed most sites as non-enhancers, although some samples showed a depletion of these marks (e.g. AML3). (b) The global distribution of covered loci is the same for all samples.

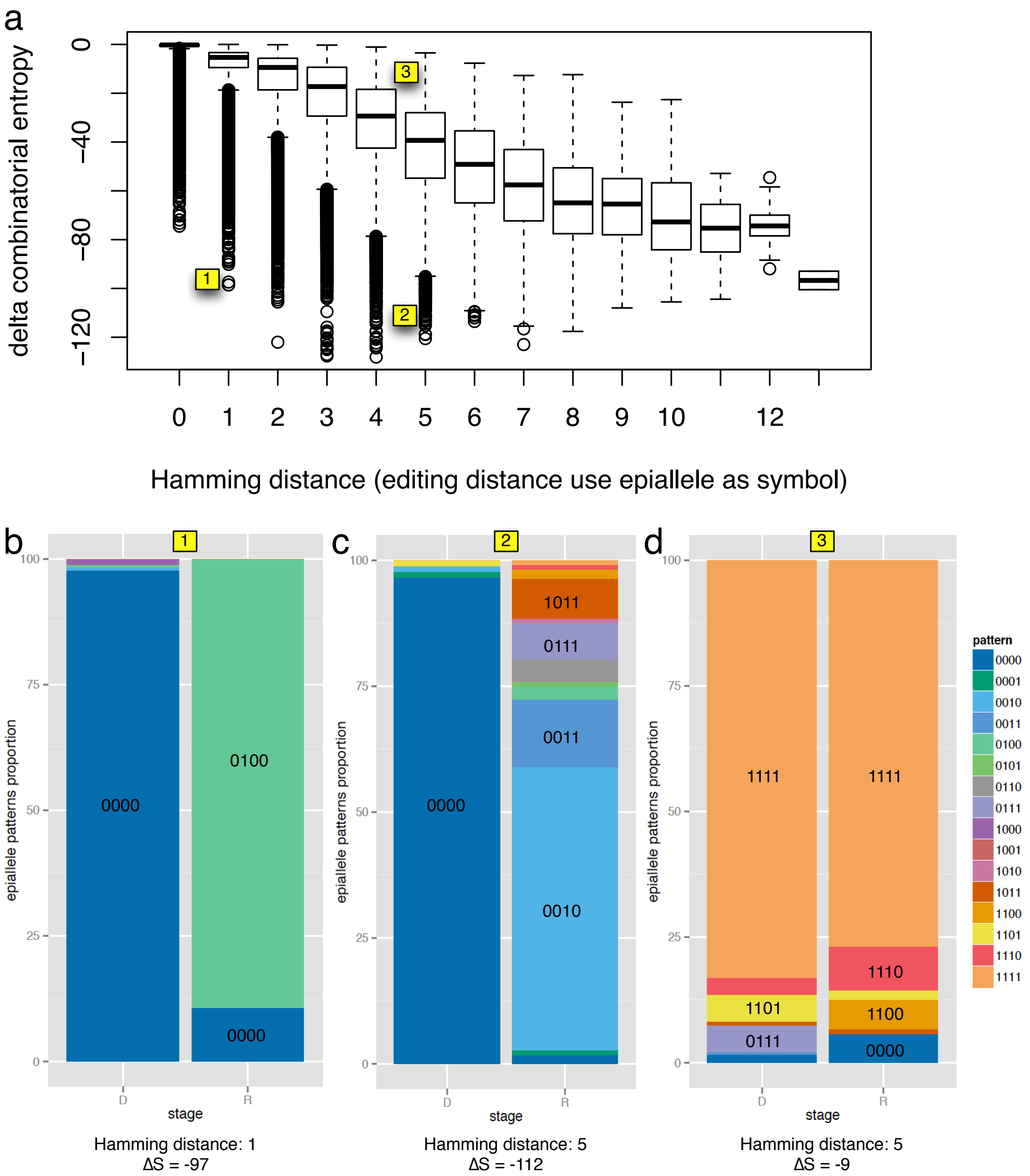

Figure S4. Hamming distance vs. delta combinatorial entropy (Δ S). (a) We used a boxplot to show, given a higher hamming distance, the lower the median ΔS. (b) Example 1 with low hamming distance (1) but high ΔS (-97). (c) Example 2 with high hamming distance (5), and high ΔS (-112). (d) Example 3 with high hamming distance (5), but low ΔS (-9). Note, hamming distance was calculated using the existence of any epiallele (> 5%) as symbol, which were marked in (b-d).

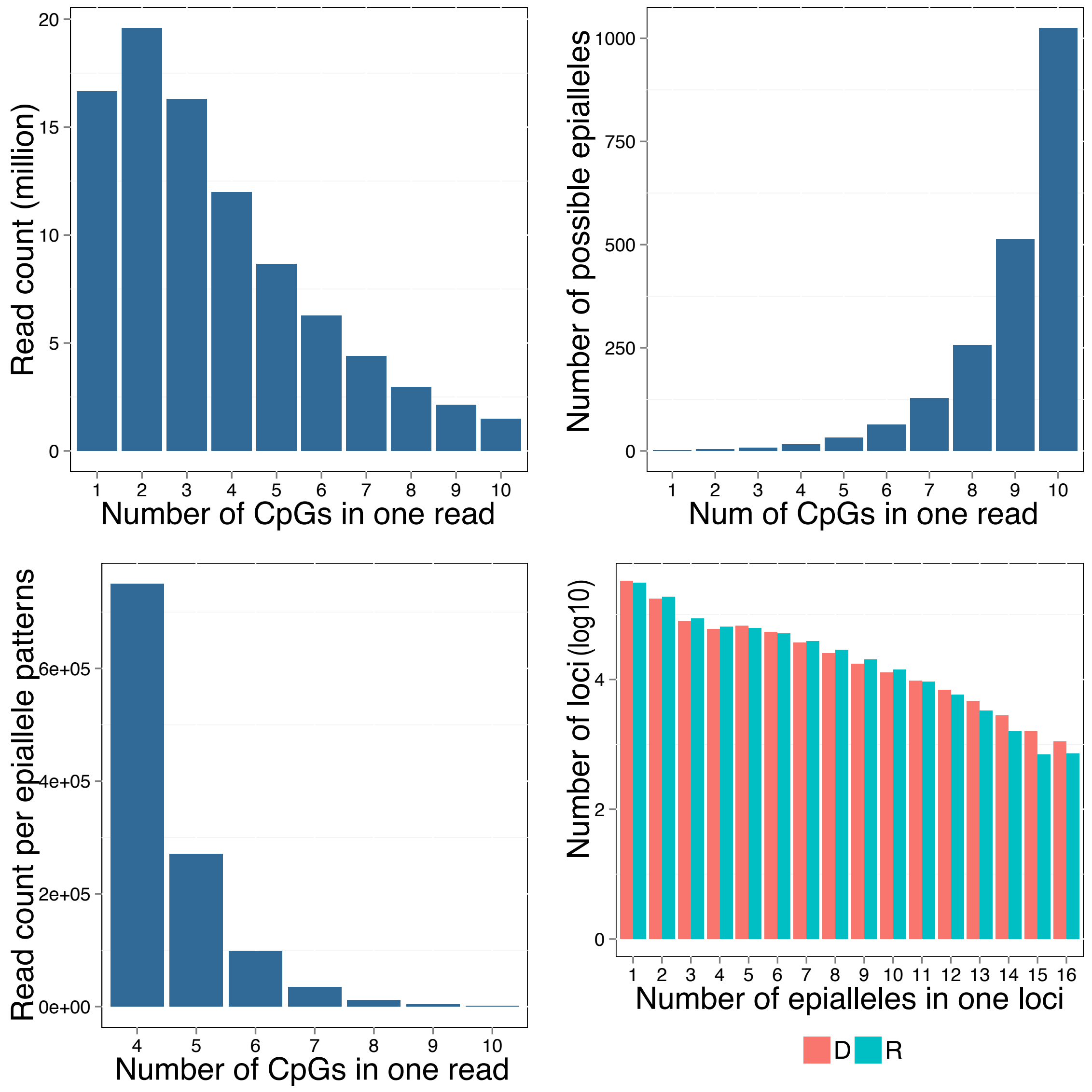

Figure S5. Coverage of CpGs by sequencing data. (a) The frequency of reads with 1 to 10 CpGs in one read. (b) The number of possible epiallele patterns with 1 to 10 CpGs in one read. (c) Read count per epiallele pattern for reads with 4 to 10 CpGs in one read. (d) The actual number of observed epialleles at each locus for diagnosis sample and relapse sample. Data from AML2 were used for calculation.

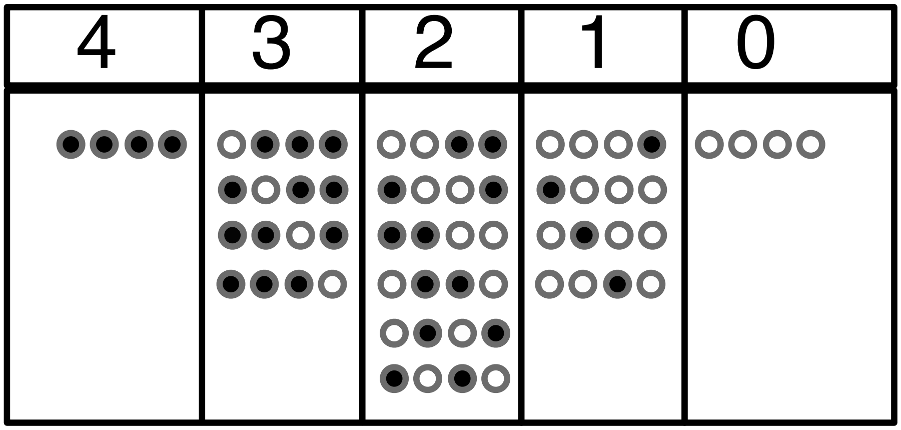

Figure S6. 16 patterns of epialleles defined by 4 CpGs within one read. This schematic shows the 16 possible states of epialleles, given the number of methylated CpGs (1,2,3,4) out of four possible (top). Filled circles represent methylated CpGs; open circles represent un-methylated CpGs.

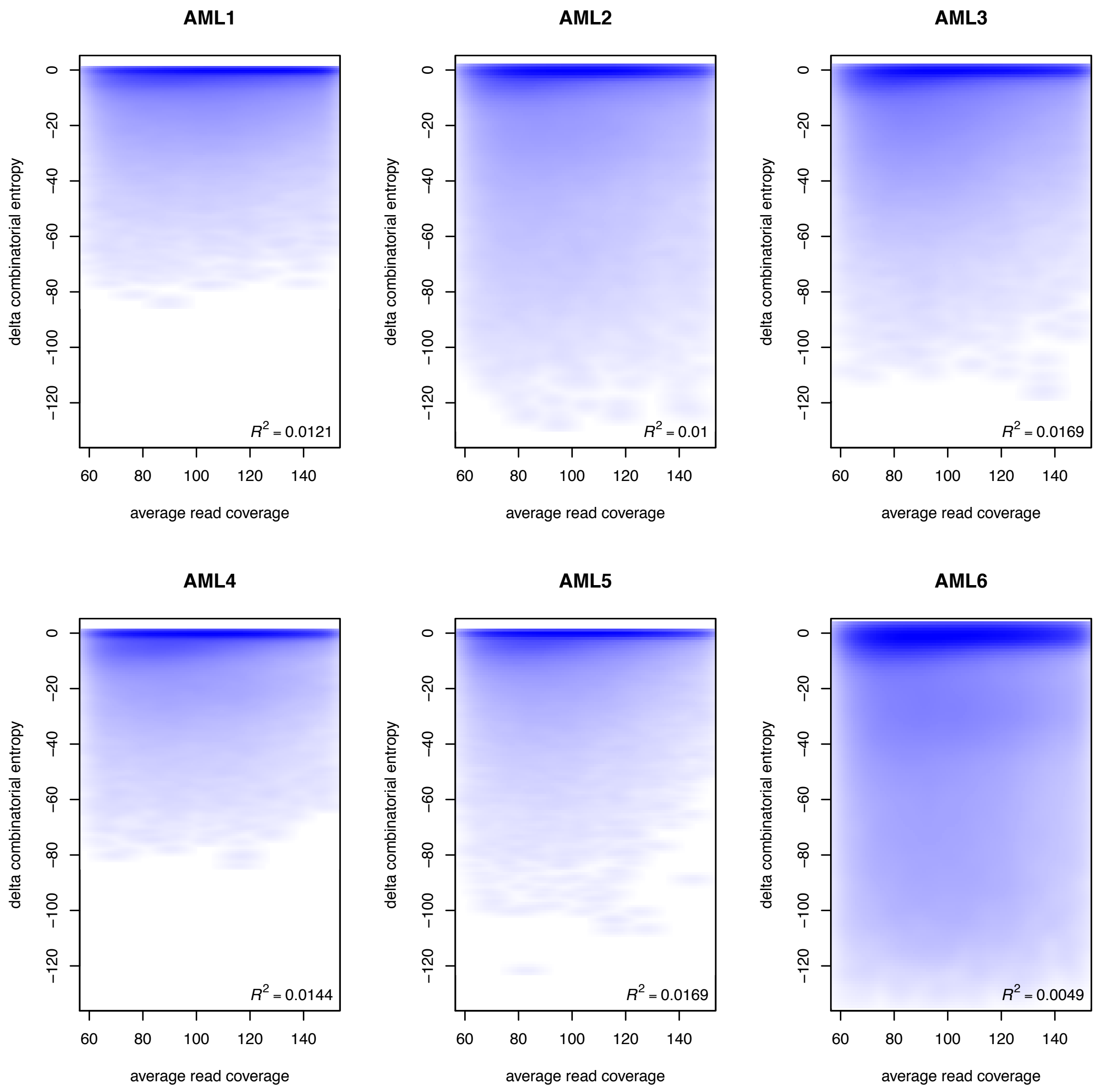

Figure S7. Average read coverage against delta combinatorial entropy. We plotted a density plot of all points showing the average coverage (x-axis) of the eloci with varying ΔS (y-axis), which showed no strong correlation (Pearson, R^2).

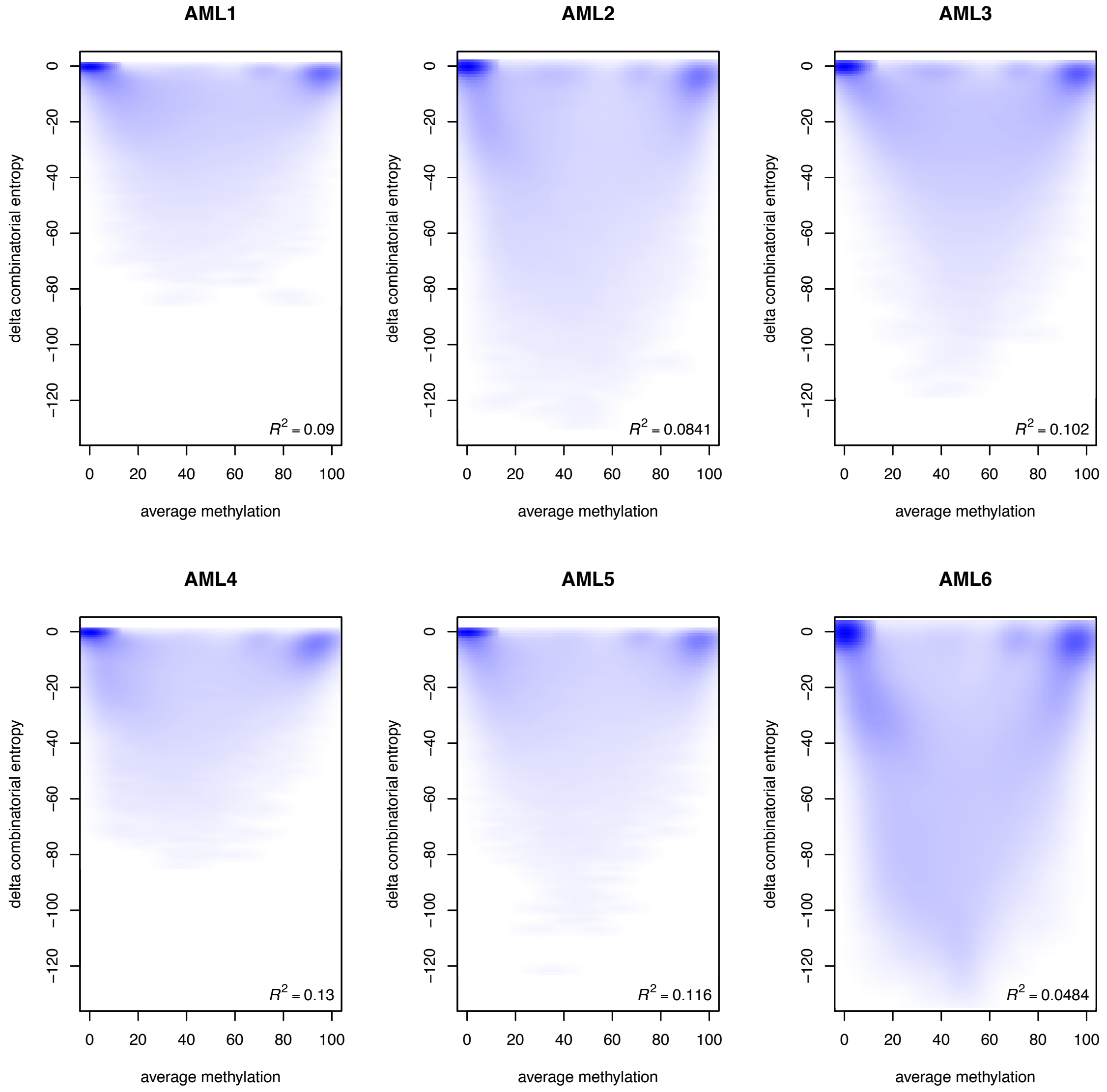

Figure S8. Average DNA methylation against delta combinatorial entropy. We plotted a density plot of all points showing the average methylation (x-axis) of the eloci with varying ΔS (y-axis), which showed a no strong correlation (Pearson, R^2). As expected, semi-methylated loci show a greater changes of being called a eloci, since there are more directions for the change to proceed (up and down).

Supplementary Table 1. Clinical information for AML patients.

ID	Sex	Age	FAB*	Death Time	Relapse Time
AML1	M	62	M4	402	266
AML2	F	50	M2	1469	637
AML3	F	67	biphenotypic	967	604
AML4	M	54	M2	N/A	939
AML5	M	62	M4	169	110
AML6	M	18	M2	N/A	606

*FAB: AML classifications use French-American-British (FAB) system.
